# Supplementary material for: The Combined Effects of Azoxystrobin and the Biosurfactant-Producing Bacillus sp. Kol B3 against the Phytopathogenic Fungus Fusarium sambucinum IM 6525
Source: Int J Mol Sci. 2024 Apr 10;25(8):4175. doi: 10.3390/ijms25084175 (PMC11049953; doi:10.3390/ijms25084175)
Supplement: Supplementary file 1 [file ijms-25-04175-s001.zip › ijms-2919033-supplementary.pdf]

Table S1. Identification of bacterial strain Kol B3 based on the similarity of the *tuf* gene sequence to the sequences stored in the NCBI database.

Bacterial strain	Sequence length [bp]	The genus/species of bacteria with the greatest similarity to the NCBI sequence	NCBI Sequence No.	Degree of Similarity [%]	Identification
Kol B3	720	*B. velezensis*	CP031694.1	100	*Bacillus* sp.
		B. amyloliquefaciens	CP014700.1	100	
		B. methylotrophicus	LN999829.1	100	

Table S2. The influence of AZ on *Bacillus* sp. Kol B3 bacteria growth.

Cultivation variant	Dried biomass [mg]	Bacterial CFU
Kol B3 (control)	21 ± 2	$10.03 \pm 5.95 \times 10^8$
Kol B3 + AZ	22 ± 2	$11.53 \pm 7.01 \times 10^8$

Kol B3 (control) – biotic control of *Bacillus* sp. Kol B3; Kol B3 + AZ – cultivation of *Bacillus* sp. Kol B3 amended with azoxystrobin.

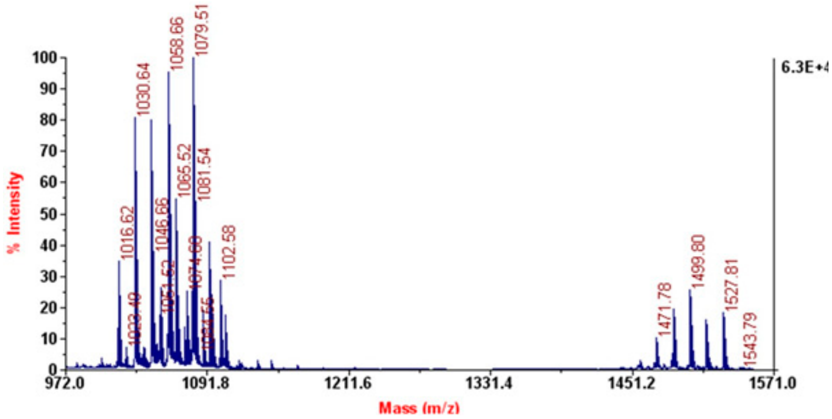

Figure S1. MALDI-TOF/TOF mass spectrum of the lipopeptide mixture presented in an extract prepared by a modified QuEChERS procedure from the culture supernatant of *Bacillus* sp. Kol B3.
